# Epoxy/alumina composite coating on welded steel 316L with excellent wear and anticorrosion properties

**DOI:** 10.1038/s41598-021-91741-y

**Published:** 2021-06-21

**Authors:** Maryama Hammi, Younes Ziat, Zakaryaa Zarhri, Charaf Laghlimi, Abdelaziz Moutcine

**Affiliations:** 1 Laboratory of Materials, Nanotechnologies and Environnement, Department of Chemistry, Faculty of Sciences, University of Mohammed V-Rabat, BP1014, Rabat, Morocco; 2 Laboratory of Engineering and Applied Technologies, Higher School of Technology, Sultan Moulay Slimane University, Beni Mellal, Morocco; 3 CONACYT-Tecnológico Nacional de México/I.T. Chetumal, Insurgentes 330, 77013 Chetumal, QR Mexico; 4 Applied Chemistry Team, Faculty of Sciences and Techniques of Al Hoceima, Abdelmalek Essaadi University, Al Hoceima, Morocco; 5 Molecular Electrochemistry and Inorganic Materials Team, Beni Mellal, Faculty of Science and Technology, Sultan Moulay Slimane University, Beni Mellal, Morocco

**Keywords:** Electrochemistry, Materials chemistry, Engineering, Materials science

## Abstract

The main purpose of this study is to elaborate anticorrosive coatings for the welded steel 316L, since this later is widely used in industrial field. Hence, within this work we have studied the electrochemical behaviour of different zones of the welded steel 316 in 1 M HCl media. The macrography study of the welded steel has revealed the different areas with a good contrast. We have stated three different zones, namely; melted zone (MZ), heat affected zone (HAZ) and base metal zone (BM). Impedance studies on welded steel 316L were conducted in 1 M HCl solution, coating of Epoxy/Alumina composite was applied on different zones, in order to reveal the anti-corrosion efficiency in each zone. Scanning electron microscopy (SEM) analysis was undertaken in order to check how far the used coating in such aggressive media protects the studied zones and these findings were assessed by water contact angle measurements. The choice of this coating is based on the cost and the safety. We concluded that the Epoxy/Alumina composite has a good protecting effect regarding welded steel in aggressive media.

## Introduction

316L welded steel is very useful in industry, infrastructure transportation and container transportation. The welded steel protection is usually fulfilled through its corrosion inhibition whose efficiency is based on many options and parameters, these parameters are related to the environmental perspective and reasonable cost. In the same framework, many studies have taken place^1–8^. For low carbon steel, the inhibitory effect of inorganic glass composed of (50% K_2_O, 25% P_2_O_5_ and 25% CaO) in 1 M HCl shows that the optical structural analysis provides a good protection for the surface^1^. The experimental results show that when the forging pressure increases, the hardness of the welding interface will increase when the tensile strength decreases^9^. The electrochemical and microstructural behaviours of "A106—Gr.B" and 316L welded pipes with nickel based alloys were studied. In welded joints, the microstructure of the heat affected zone on the root weld leads to strong grain refinement and polygonal ferrite and pearlite formation by welding heat cycle change^10^.

Also, effect of the microstructure on contact angle (CA) and corrosion of ductile Iron–Graphite composite "ferrite, pearlite and graphite" was studied. It was proven that pearlite is more susceptible to corrosion than (ferrite and graphite). A higher portion of pearlite in the microstructure can be detrimental to corrosion resistance of the material^11^.

## Microstructure characterization of the steel 316L and the effect of the welding energy

### Preparation and characterization of the microstructure steel 316L

**Figure 1:**
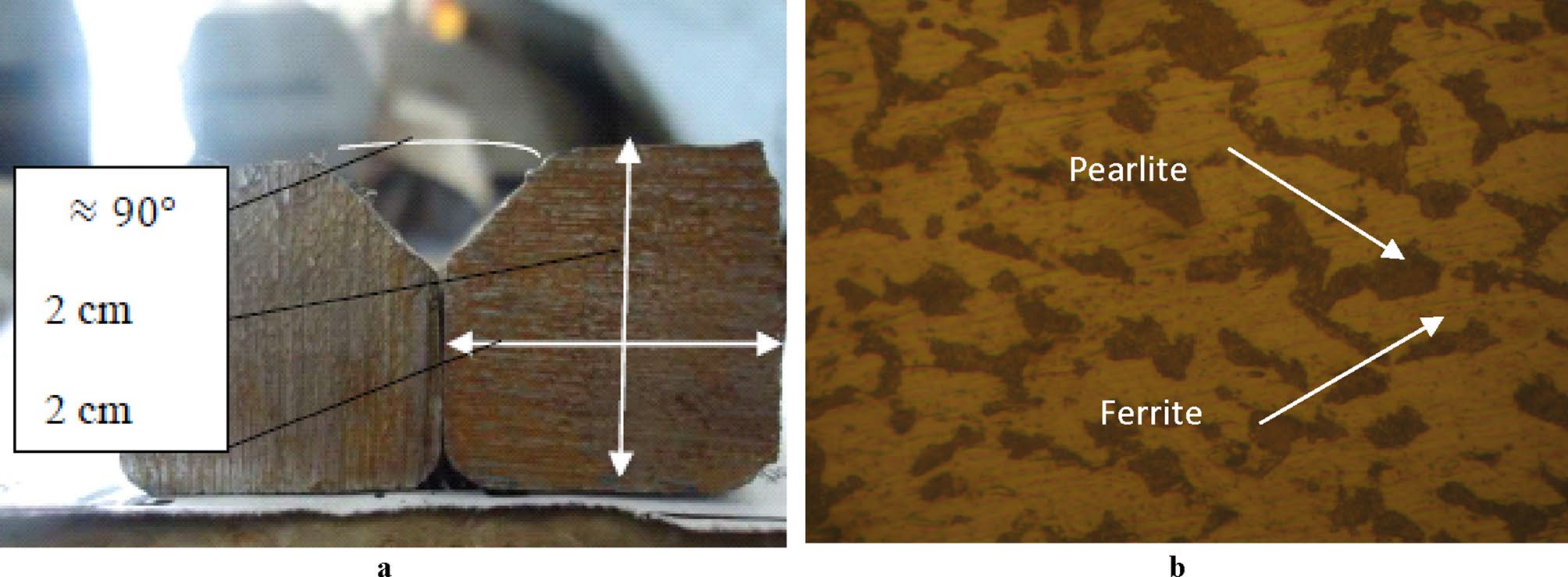
(**a**) Geometry form of the steel 316L before the welding. (**b**) microstructure of the steel 316L before the welding “magnified 300 times”.

In this section, we prepared base metals to identify the microstructure before welding. After that, we tried to highlight the influence of thermal cycle changes on the microstructure and the mechanical properties of welded steel joints with similar filler metals. We prepared welding samples by cutting a pair of ingots with a dimension of 2 cm $\times$ 2 cm (Fig. 1a).

**Figure 2:**
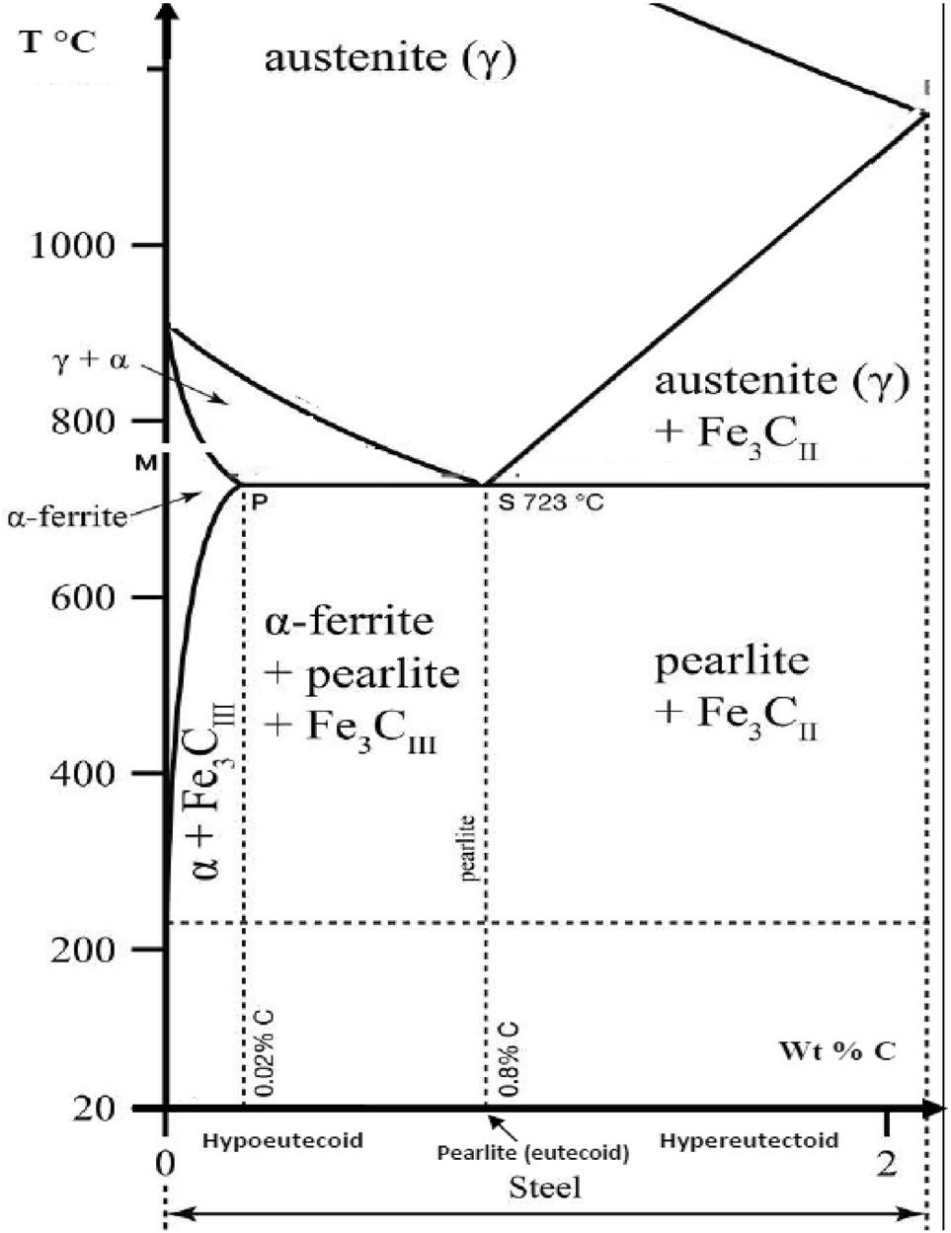
Binary diagram of the steel containing carbon below 2 wt%.

To eliminate the surfaces roughness, the steel ingots were polished by 300, 500, 800 and 1000 grain sizes abrasive papers. This step was followed by the electrolytic polishing, we prepared an acid solution based on 5% H_2_SO_4_ and 95% H_3_PO_4_. Afterward, we characterize the microstructure using an optical microscope, see Fig. 1b, where the pearlite is dark and the ferrite is yellow. The pearlite is of (10% Fe_3_C and 90% ferrite α). For the present sample, the rate of carbon is of 0.8 wt % before the welding, which is in a good consistence with the Fe–C “binary diagram”, Fig. 2, this steel is in hypoeutecoid area.

### Principle of arc welding electrodes

This process based on generating the heat by an electric arc-struck between the samples and the electrode, a simultaneously melt occurs between the electrode and metal (500 to 800 °C).

The intensity for the flat steel welding is:

1 $$ I = {\text{ K }}(\varphi - {1}) \left[ {\text{A}} \right] $$

K is a constant and stands for the generator, K = Cte = 50.

2 $$ {\text{I}}_{{{\text{max}}}} = {\text{ K}}.\varphi {\text{ and }} \varphi = {\text{diameter of the electrode }}\left[ {{\text{mm}}} \right] $$

The welding energy is the displacement of heat source per length unit of the weld:

3 $$ {\text{E}} = \frac{U\left( V \right) \times I\left( A \right)}{{{\text{V}}\left( \frac{cm}{s} \right)}}\left[ \frac{J}{cm} \right] $$

where the dissipated energy is expressed by:

4 $$ E_{d} = E.\eta $$

and *η* stands for the yield.

The equivalent energy is:

5 $$ E_{eq} = E.\eta .k $$

*k* is a correction coefficient connected to the geometry of the joint to be welded, Fig. 1a, for the joint in Y with Y *≈ 90°* (*k* = 0.67). In the current study, the used welded energy is of 20,183.58 $\rm{J}.{cm}^{-1}$

### Welded sample characterization

**Figure 3:**
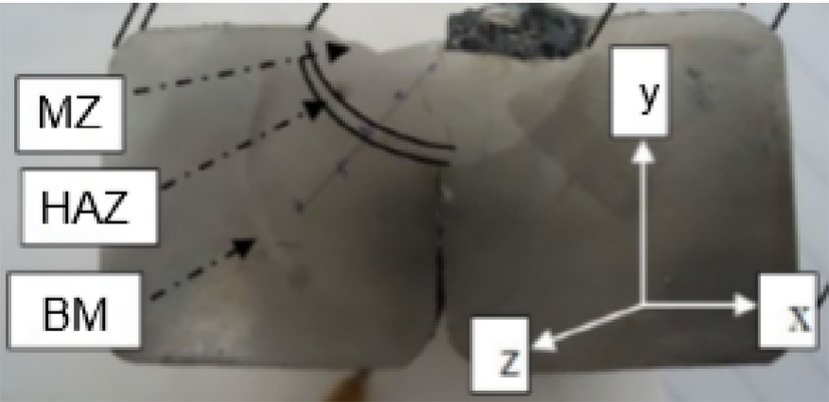
Macrographic observation.

The Fig. 3, on the (XY) plan, shows different areas noted base metal (BM), heat affected zone (HAZ) and melted zone (MZ).

To study the microstructure, the optical microscope is a basic method. For the preparation of the steel 316L, it must to avoid any structural modification due to the hardening and heating of the metal.

**Figure 4:**
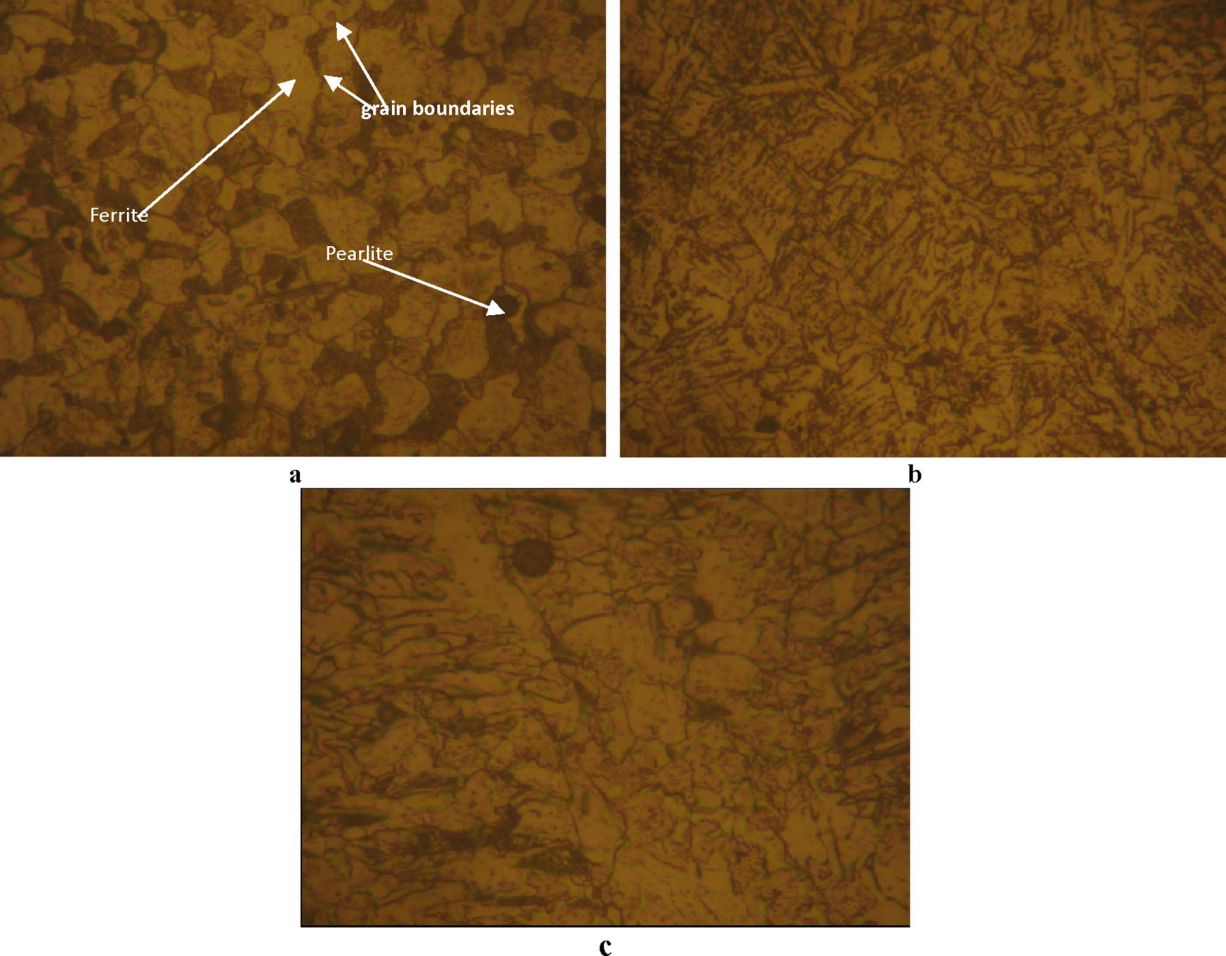
Microstructure of the welded steel 316L [magnified 300 times]. (**a**–**c**) from MZ, HAZ and BM zones; respectively.

From the base metal, Fig. 4a compared to Fig. 1b, the grain boundaries appear and the sizes of grains begin to decrease due to the thermal cycling of the welding energy.

In the heat affected zone, Fig. 4b, the observation shown the grains elongation where the directions of the grains are not the same compared to Fig. 4a. The melted zone microstructure of the Fig. 4c is different compared to the microstructure of the HAZ, interpreted by solidification aspect, composed of two phases austenite (γ) and ferrite (α). Then, the energy of the welding affected the grain size. Also, the orientation depends on the direction of cooling.

### Microstructure and the mechanical properties

The hardness of the steel 316L defines the resistance of a surface of the sample to the penetration of a harder body using a tip or ball of a durometer. There are many hardness tests to estimate or compare the forte or robustness of materials^12–13^.

For this, we can use the Vickers hardness principle to measure the resistance of the different samples; according to the following relation^14–15^:

6 $${\rm{H}}_{\rm{V}}=2{\rm F}.\frac{{\rm sin}\left(\frac{136^\circ }{2}\right)}{\rm{g}.{\rm{d}}^2}.$$

Then ${\rm{H}}_{\rm{V}}\approx \frac{0.189.\rm{F}}{{\rm d}^2}$ where, ${\rm{H}}_{\rm{V}}$: Vickers hardness, F: Applied force [N], d: footprint diagonals [mm], g: Gravitational acceleration [${m/s}^{2}$].

**Figure 5:**
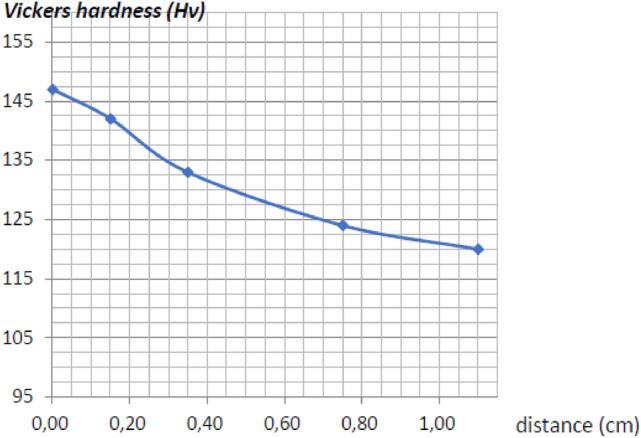
Vickers hardness values in terms of the distance on the welded sectional plan XY (from MZ to BM).

For the welded sample, we find that the hardness decreases from MZ to BM, see Fig. 5. This can be explained by the thermal fact of the ${E}_{d}$ “energy dissipated” on the microstructure of the grains and joints. The structural reorganizations depend on the impact of the welding heat flux. The hardness of the present steel is being higher where its grains are being finer too^16–17^. The elastic limit depends on the inverse of the square root of the “d” grain size associated to the Hall Petch expression:

7 $$\upsigma ={\upsigma }_{0}+\frac{k}{\sqrt{d}}$$

(${\upsigma }_{0}$ and k) are constants of the material.

## Corrosion behaviour of the three studied zones

There are numerous methods to study the corrosion, most of them use different physicochemical properties of the concerned samples. It is often important to make a qualitative assessment of the corrosion type; this requires simple visual observation. Depending on the type and the conditions of the involved corrosion. The quantitative corrosion assessment can use different methods. Let us quote:

- Thickness measurements or gravimetric method.
- Mechanical resistance measurements (traction, compression, torsion, hardness, etc.).
- Chemical analysis of the corroding medium, in particular by electrochemical methods of solution analysis, such as polarography and electrochemical methods

Within our study we are particularly interested to the electrochemical methods which allows us following the corrosion behaviour and coating protection effect of the three studied zones of the welded steel 316L; namely melted zone (MZ), heat affected zone (HAZ) and base metal zone (BM).

### Electrochemical measurements

Electrochemical experiments are carried out in a thermostated, double-walled pyrex cell, coupled with a conventional assembly with three electrodes:

- The welded steel sample as working electrode;
- A platinum electrode as counter electrode;
- A saturated calomel electrode (SCE) as reference electrode.

Each of the studied zone of the welded steel is regarded as a working electrode. The latter is in the form of a disc where only an active part of 0.8 cm^2^ is exposed to the aggressive solution, the rest is covered with resin. The electrochemical measurements are carried out with an assembly comprising a potentiostat Tacussel, Radiometer PGP 201 type, piloted by the software Voltamaster1. The intensity-potential curves or polarization curve of the metal /solution interface are obtained in potentiodynamic mode; the potential applied to the sample varies continuously from − 750 to + 750 mV vs SCE, with a scanning speed of 30 mV/min. The intensity of the current is measured between the working electrode and the counter electrode.

**Figure 6:**
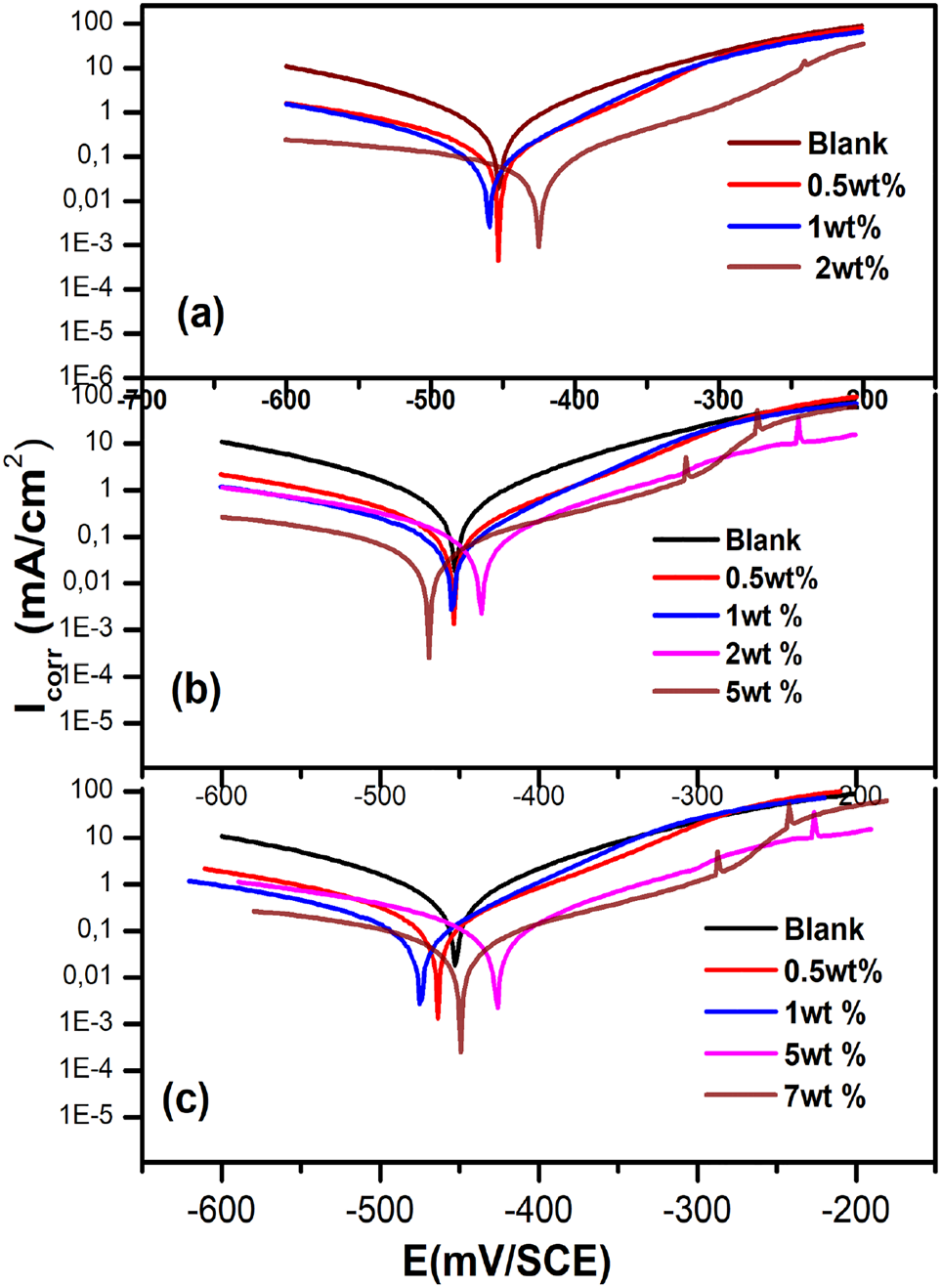
Polarization curves for steel 316L in 1 M HCl medium without and with Epoxy/Alumina coating at different concentrations of incorporated alumina corresponding to (**a**) Base metal zone, (**b**) Heat affected zone and (**c**) melted zone.

According to the polarization curves which are exhibited in Fig. 6, it can be noticed that the weight percentage of alumina added to the epoxy resin reduces the current density of the cathode and anode, which means that the inhibitory efficiency of the coating is of mixed type. The decrease in current density is caused by the low Alumina concentration “2% (weight)” in the base metal. At the same concentration, the heat affected zone always has a larger current density, whereas, melted zone, the current density is greater for the same percentage of alumina.

The results showed the decrease in ${\rm{I}}_{\rm{corr}}$ with the coating as compared to blank solution, this is probably due to the increase adhesion of the alumina reinforced coatings on metal surface which increases inhibition efficiency. The corrosion current density (${\rm{I}}_{\rm{corr}}$) can be obtained by extrapolating the Tafel lines to the corrosion potential. The inhibition efficiency ($\rm{IE}(\rm{\%})$) values were calculated from the relation^1^:

8 $$\rm{IE}\left(\rm{\%}\right)=\frac{{\rm{I}}_{\rm{corr}}^{0}-{\rm{I}}_{\rm{corr}}}{{\rm{I}}_{\rm{corr}}^{0}}\times 100$$

where ${\rm{I}}_{\rm{corr}}^{0}$ stands for the corrosion current density extracted in the blank solution while ${\rm{I}}_{\rm{corr}}$ denotes for the current measured in the presence of coating.

Table 1 gathers the values associated with the electrochemical parameters, which are determined from the curves as well as the inhibitory efficiency. The corrosion current density decreases as the alumina concentration increases and therefore E% increases. The composite containing 7wt% of alumina is therefore considered to be the best protective film in the welded area whose inhibitory efficiency is maximum and reaches 95%.

**Table 1:** Polarization parameters and coating efficiency values for base metal zone, heat affected zone and melted zone.

Studied zone	wt% of Alumina	i(µA/cm2)	E (%)
Base metal zone (BMZ)	0.5wt%	256	59
	1wt%	125	80
	2wt%	25	91
Heat affected zone (HAZ)	0.5wt%	200	78.68
	1wt%	96.54	82.96
	2wt%	83	86.63
	5wt%	55.47	90.06
Melted zone (MZ)	0.5wt%	83	84.82
	1wt%	74	86.79
	5wt%	50	91.33
	7wt%	29.68	95.34

### Electrochemical impedance spectroscopy

**Figure 7:**
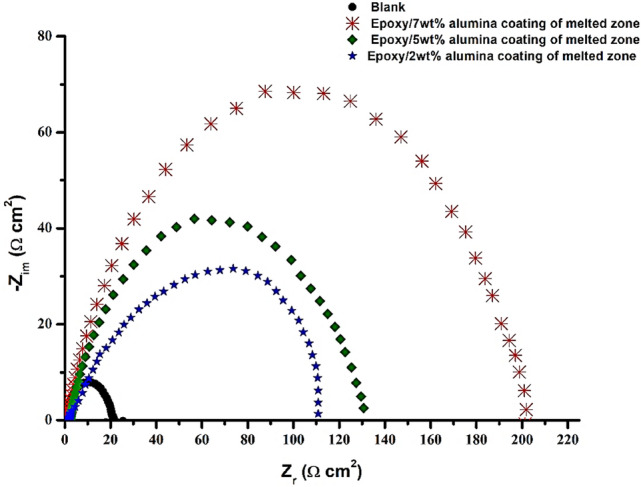
Nyquist diagrams of melted zone in 1 M HCl without and with coating containing various concentrations of alumina.

Electrochemical impedance spectroscopy measurements were carried out in order to study the kinetics of the electrode process and the surface properties of the studied system. This method is widely used to investigate the corrosion inhibition process^18–19^. Nyquist plots of steel specimens in 1 M HCl solution in the absence and presence of coating with the optimum concentrations of alumina related to each area are shown in Fig. 7. A high frequency depressed charge transfer semicircle is observed^20–21^. It is clear from Fig. 7 that the impedance spectra are not perfect semicircles and the depressed capacitive loop corresponds to surface heterogeneity which may be the result of surface roughness or dislocation. The inhibition efficiency is calculated using charge transfer resistance (${\rm{R}}_{\rm{ct}}$) as follows^22–23^:

9 $$\rm{IE}\left(\rm{\%}\right)=\frac{{\rm{R}}_{\rm{ct}\left(\rm{inh}\right)-}{\rm{R}}_{\rm{ct}}}{{\rm{R}}_{\rm{ct}(\rm{inh})}}\times 100$$

where, ${\rm{R}}_{\rm{ct}\left(\rm{inh}\right)}$ and ${\rm{R}}_{\rm{ct}}$ are the values of charge transfer resistance in presence and absence of Epoxy/ Alumina coating in 1 M HCl solution, respectively.

In the Epoxy/Alumina coating, the increase in the concentration of alumina induces the increase in the diameter of the loops as shown in the Nyquist diagrams (see Fig. 7), which suggests that the inhibition efficiency is improved with increasing concentration alumina coating. The results obtained are in good agreement with the potentiodynamic polarization curves.

The inhibition efficiency (EI%) obtained from the electrochemical impedance spectroscopy results also increases with the increase in the concentration of alumina, see Table 2.

**Table 2:** charge transfer resistance of melted zone with epoxy/alumina coating in 1 M HCl solution and their efficiency (EI%).

Alumina (wt%)	R_C_ (Ω.cm^2^)	E%
2wt%	111	80.18%
5wt%	132	83.3%
7wt%	201	89%

The values obtained are consistent with those calculated using potentiodynamic polarization measurements.

**Figure 8:**
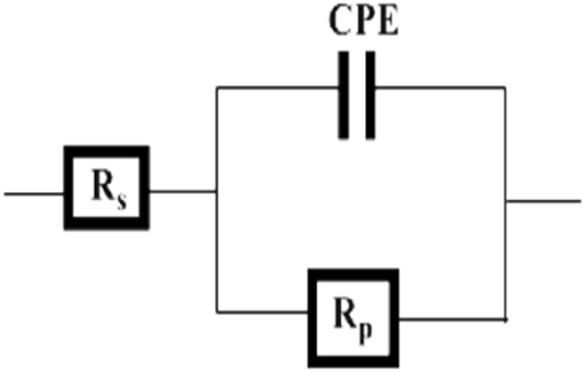
Equivalent circuit for welded steel uncoated and coated with Epoxy /Alumina composites at different concentration in 1 M HCl solution.

The equivalent circuit (EEC) is illustrated in Fig. 8 without and with various concentrations of Epoxy /Alumina coating employed, respectively. The EEC is composed of the solution resistance (Rs), polarization resistance (Rp) and constant phase element (CPE). This equivalent circuit is similar to the ones obtained by different researchers^24–31^.

### SEM/EDX characterization

**Figure 9:**
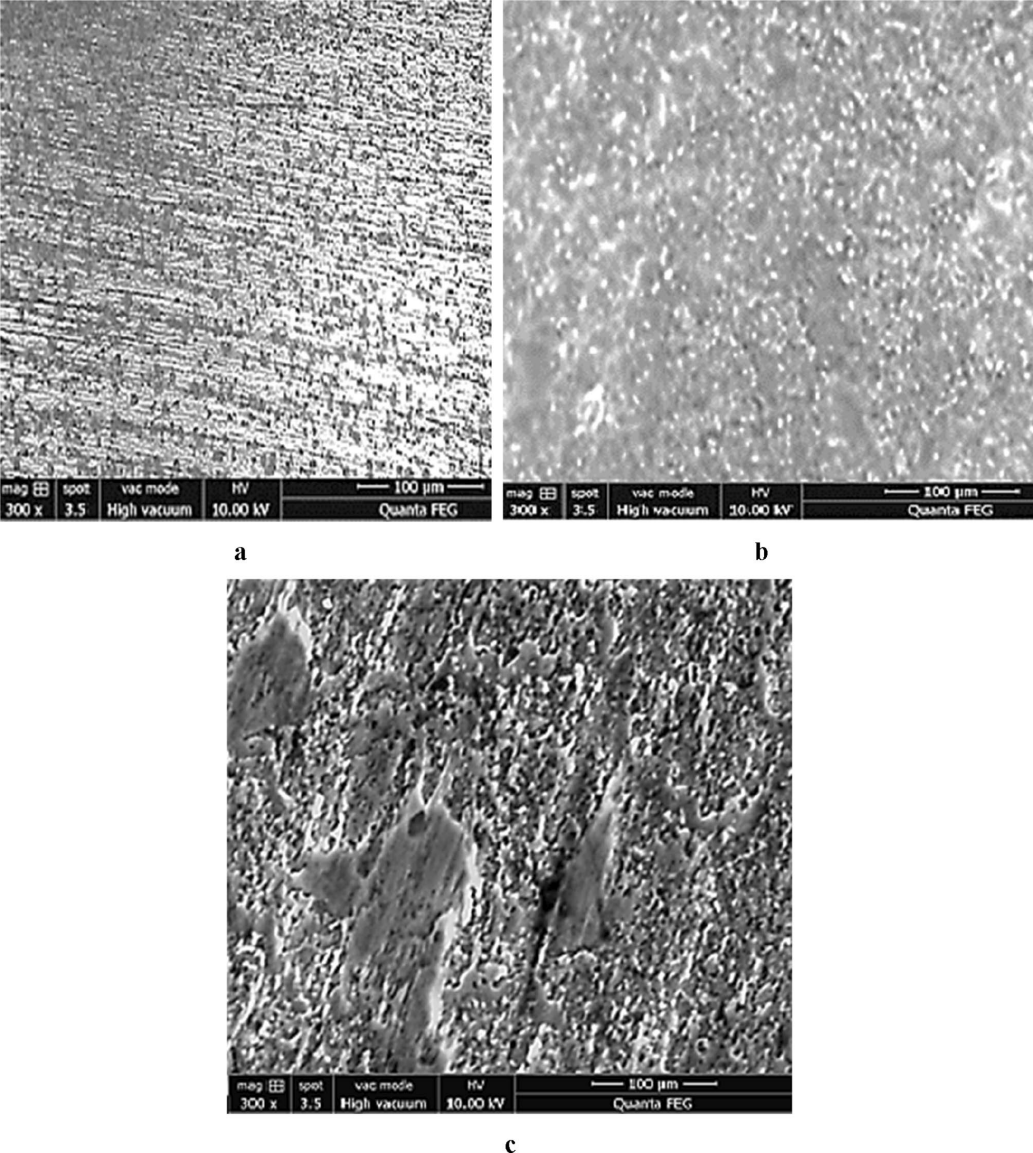
SEM micrographs of attacked zones; (**a**): base metal zone, (**b**): Heat affected zone and (**c**): melted zone.

In order to obtain further findings on the properties of welded steel with and without the optimal concentration of alumina in the Epoxy/Alumina coating in 1 M HCl medium, we conducted a morphological study using SEM. Figure 9 shows SEM micrographs of three studied areas with the same magnification, revealing the state of each area after being immersed in the corrosive solution at 303 K for 1 h. According to the SEM image, obtained after welding the steel strip exposed in the HCl solution, it can be observed that the surface is very damaged and rough, which means that the corrosion product covers the surface of the welded steel. In addition, due to the use of abrasive paper for polishing treatment, some scratches appeared on the steel surface.

Compared with SEM micrographs of other areas, the SEM image captured for the melted area is exposed to corrosion, shows that the surface is very etched and rough. This observation is probably the result of corrosion product formation. However, we can clearly observe that the corrosion on the entire inspection surface is not uniform.

**Figure 10:**
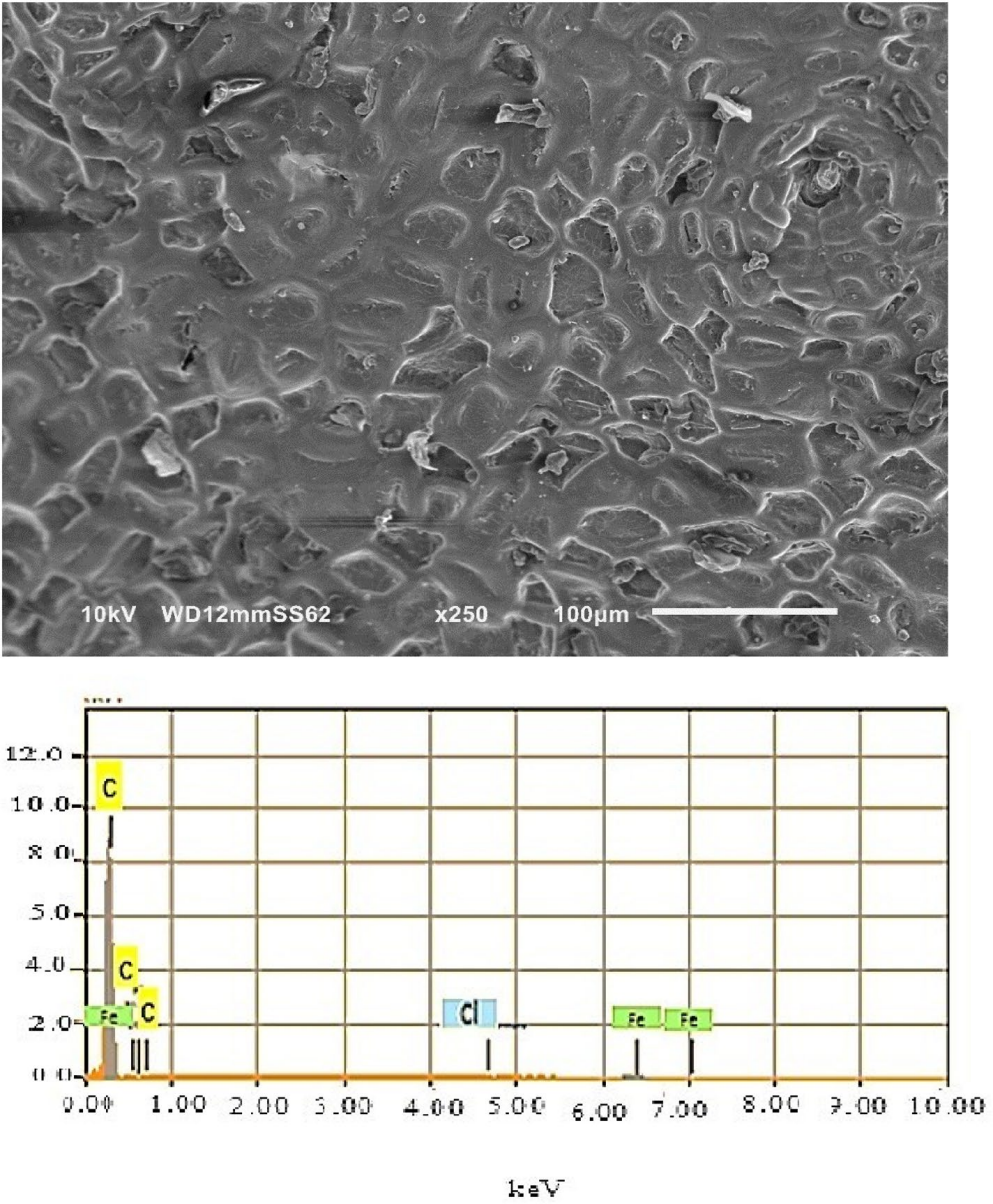
SEM analysis along with EDX of coated melted zone.

**Figure 11:**
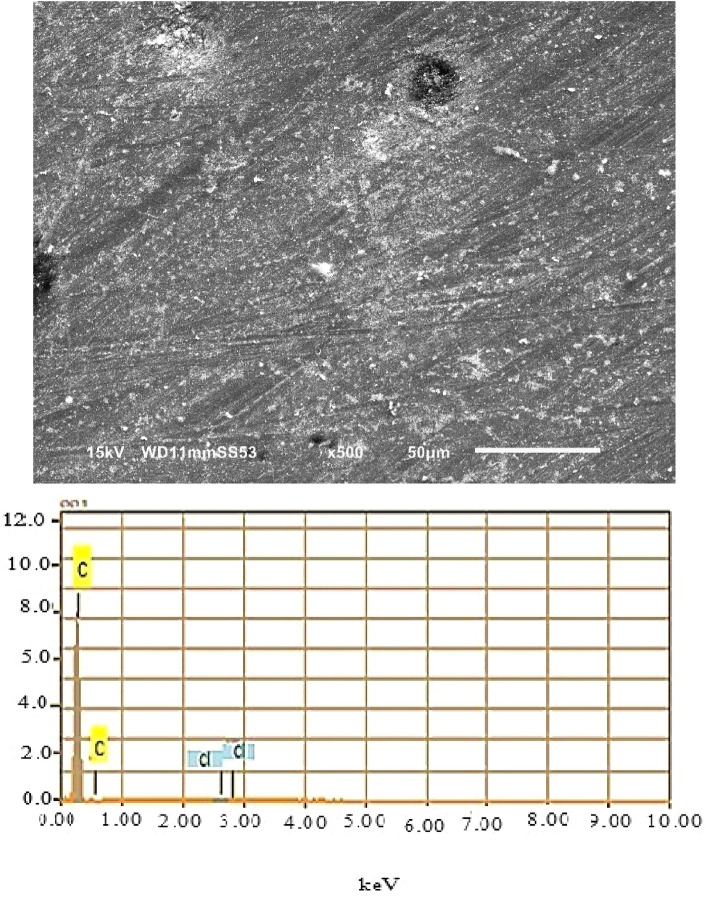
SEM analysis along with EDX of coated heat affected zone.

**Figure 12:**
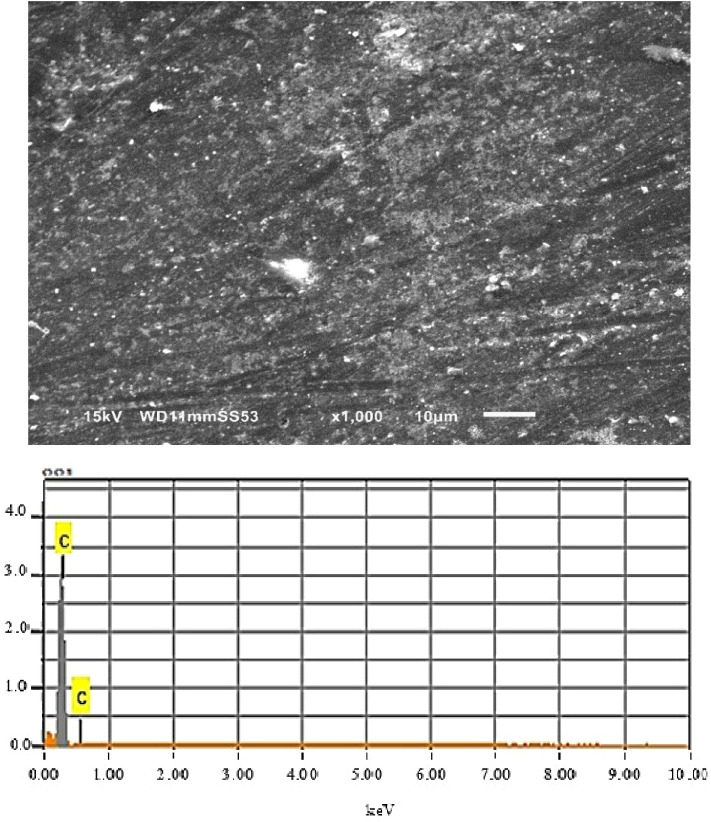
SEM analysis along with EDX of coated base metal zone.

In order to examine the coated zones, analyzes of the morphology coupled with EDX spectra were performed on coatings of different zones of surfaces, see Figs. 10, 11 and 12. The SEM micrographs of the coated zones (melted zone, heat affected zone and base metal zone), note that the coating composition of the three zones is the same (Epoxy/2wt%Alumina).

As depicted in Fig. 10, the SEM image captured after exposure of the melted zone in 1 M HCl solution show that this zone surface is not completely covered, because of the existence of some pores.

The EDX analysis evaluates the SEM observations and indicates signals attributed to Fe and a small fraction of Cl from the solution was also detected, this means that the applied coating composition is not perfectly protecting the melted zone surface. However, by comparison with the image obtained without coating we can conclude that the surface of melted zone is almost free from corrosion in the presence of coating. This is due to the formation of a physical barrier on the surface of the melted zone. These observations show that Epoxy/2wt% Alumina coating prevents corrosion of melted zone by limiting access of the electrolyte to the surface.

According to SEM images of heat affected zone in the presence of the coating as illustrated in Fig. 11, the surface is almost free of corrosion products. This suggests that corrosion processes are slowed or stopped by the coating. But the protection is not yet perfect because of the existing of small pores on the coating surface.

EDX analysis of the electrode surface revealed peaks with small signals Cl suggesting that the surface is protected by the coating but due to the existence of small pores Cl of the aggressive solution could reach to the studied surface.

The SEM micrograph of the coating of the base metal zone is exhibited in Fig. 12. It shows that the surface is completely covered and this observation was assessed by EDX analysis which indicated only carbon peaks. Therefore, the base metal zone is considered perfectly protected.

By comparison of the results obtained in the three studied areas (melted zone, heat affected zone and base metal zone), we find that the rate of dissolution varies according to the following direction:

$${\text{Base metal zone}}<{\text{heat affected zone}}<{\text{melted zone}}$$

The inhibition efficiency obtained in the three studied media after 1 h of immersion also follows the same order, which is in good agreement with the results obtained through polarization and impedance. This is completely consistent with the observation results of uncoated and coated SEM images.

As a recapitulative, the coatings are studied through surface topography and electrochemical techniques. The Nyquist diagrams of coatings with different weight percentages of alumina content show that the Rct value increases, thus increasing inhibition efficiency. The potentiodynamic curves show that the coating is actually a mixed type. Surface analysis by scanning electron microscope also proved the results obtained by electrochemical experiments.

### Contact angle experiments

The wettability of the uncoated and coated surfaces was investigated via measurement of the contact angle by placing a water droplet in contact with the surface using a micrometer syringe. To measure the contact angle, a camera was used to scan the droplet profile, furthermore, to avoid the effect of weight, the drop size of the distilled water was about 3 μL.

In order to check that the coating has protective effect on the studied surfaces, we measured the contact angle on the attacked surfaces, which were immersed in 1 M HCl solution without and with coatings.

**Figure 13:**
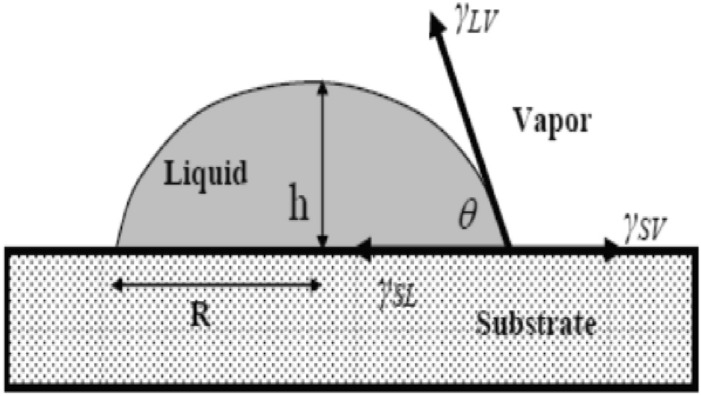
Principle of contact angle calculation.

The wettability of our elaborated samples has been determined using the process of Owens–Wendt^32^. The image of droplet (Fig. 13) was taken with external CCD camera connected to the computer.

Then, the angle θ was calculated from the dimensions of the picture droplet, using the Analyzing Digital Images (software developed and freely distributed by STEM Education Institute, University of Massachusetts Amherst in USA) and the following relation:

10 $$ \theta = 2{\text{Arctg}} {{(2h/d)}} $$

with: θ < 90, where h and d stand for the droplet’s height and length, respectively.

For more precision, the angle θ was also directly measured through recorded image with high-resolution CCD camera of the liquid droplet profile sited on the samples using screen protactor version 3.2 software of Iconico Inc., New York, NY, USA.

**Figure 14:**
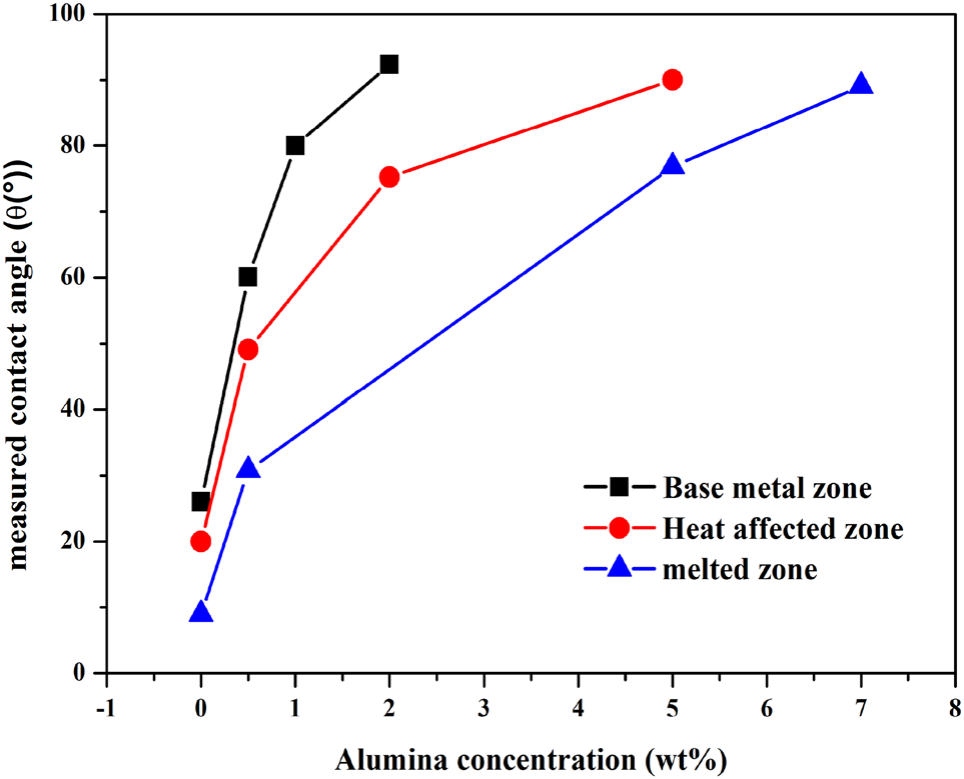
Evolution of contact angle values on the three studied zones without and with Epoxy/wt% Alumina coatings.

According to the curve depicted in Fig. 14, we noticed that the uncoated surface (0 wt%) showed very low contact angle values, which may be attributed to the surface roughness and pores mentioned in the SEM analysis. It is worth noting that the contact angle corresponding to the melted zone is the lowest (about 10°), and the water contact angle in the base metal zone is the highest (26°), but the overall contact angle is still very low and shows the hydrophilicity of the surface.

However, for the metal base area coated with Epoxy resin/0.5wt% Alumina, the contact angle increases sharply to 60°.

In the case of the heat affected zone coating, the contact angle is 45°, and the lowest value is attributed to the contact angle is on the coating melting zone (is of 26°). This may be due to the strengthening effect of the alumina content on the epoxy matrix. In fact, the surface of the steel strip was studied in 1 M HCl solution. Compared with the uncoated surface, the contact angle of the coating increased significantly. This is probably due to the good adhesion of the Epoxy/Alumina coating to the surface.

The contact angle is the balance between adhesive and cohesive energies. The cohesive surface energy of liquid (W_c_ = 2γ_L_) due to the van der Waals attractive intermolecular forces; dispersive (London), polar forces (Keesom, Debye), or hydrogen bonds^33^ can be stronger than the force of adhesion attraction between the liquid molecules and the atoms in the solid (Wc = 2_γL_ > W_SL_) and hence the liquid will minimize contact with the solid surface and form a compact liquid droplet. Hence, the wetting of the surface is unfavorable.

In order to reveal the connection between the values of the contact angles measured on the attacked surface with the protective effect of the coatings, we have calculated the adhesion energy ${w}_{sl}^{a}$ of the surface of the studied samples (Table 3).

**Table 3:** Contact angle and work adhesion W_SL_ of the coatings of the studied zones.

Zones	Sample	θ (°)	W_SL_ (mJ/m^2^)
Melted zone	Uncoated surface	10	144.508
	0.9wt%Alumina/epoxy	26	138.174
	5wt%Alumina/epoxy	72	95.295
	7wt%Alumina/epoxy	81	84.157
Heat affected zone	Uncoated surface	18	142.033
	0.5wt%Alumina/epoxy	45	124.277
	2wt%Alumina/epoxy	70	97.697
	5wt%Alumina/epoxy	85	79.134
Base metal zone	Uncoated surface	24	139.303
	0.5wt%Alumina/epoxy	60	109.200
	1wt%Alumina/epoxy	82	82.919
	2wt%Alumina/epoxy	89	74.037

The determination of the adhesion energy by the measurements of the contact angle is achieved through the following equation:

11 $$ \Delta G_{sL}^{a} = - \gamma_{L} (1 + \cos \theta ) = - W_{sL}^{a} $$

The same equation can be written as

12 $$ \cos \theta = W_{sL}^{a} /\gamma_{L} - 1 $$

This equation shows that the contact angle represents the balance competition between the adhesion energy (W^a^_sl_) and the cohesion energy of liquid (W^c^ = 2γ_L_).

In our case, the used fluid is water, with high surface tension of γ_L_ = 72.8 mJ/m^2^ or cohesion energy of Wc = 2_γL_ = 145.6 mJ/m^2^.

In conclusion of this part, the study of the wettability by water of Epoxy /Alumina composite coating showed that the loading of metallic fillers modifies the surface of materials. This effect has lowered their solid surface tension (γs), and consequently favoring the apparition of the hydrophobicity. This result is important because the coating becomes hydrophobic by loading it with alumina.

## Conclusion

The current work focuses on the microstructure coupling study of three areas of welded steel through several electrochemical and chemical methods to study the protective effect of Epoxy/Alumina coating on welded steel corrosion in 1 M HCl environment. The experimental results clearly show that the applied coating has excellent corrosion resistance and the concentration of embedded alumina associated with each area is accurate. The polarization curve shows that the coating acts as a mixed inhibitor while reducing the anodic and cathodic processes. In addition, SEM characterization shows that the Epoxy/Alumina coating prevents corrosion in three areas through a very protective film that hinders the formation of corrosion products. The protective film is also characterized by EDX, indicating that the coating is combined with the surface of the welded steel strip, resulting in hydrophobic behaviour.
